# Whole-exome sequencing reveals the mutational spectrum of testicular germ cell tumours

**DOI:** 10.1038/ncomms6973

**Published:** 2015-01-22

**Authors:** Kevin Litchfield, Brenda Summersgill, Shawn Yost, Razvan Sultana, Karim Labreche, Darshna Dudakia, Anthony Renwick, Sheila Seal, Reem Al-Saadi, Peter Broderick, Nicholas C. Turner, Richard S. Houlston, Robert Huddart, Janet Shipley, Clare Turnbull

**Affiliations:** 1 Division of Genetics and Epidemiology, The Institute of Cancer Research, Fulham Road, London SW3 6JB, UK; 2 Divisions of Molecular Pathology and Cancer Therapeutics, The Institute of Cancer Research, Fulham Road, London SW3 6JB, UK; 3 Inserm U 1127, CNRS UMR 7225, Sorbonne Universités, UPMC Univ Paris 06 UMR S 1127, Institut du Cerveau et de la Moelle épinière, ICM, F-75019, Paris, France; 4 The Breakthrough Breast Cancer Research Centre, The Institute of Cancer Research, Fulham Road, London SW3 6JB, UK; 5 Academic Radiotherapy Unit, The Institute of Cancer Research, Fulham Road, London SW3 6JB, UK; 6 William Harvey Research Institute, Queen Mary University London, Charterhouse Square, London EC1M 6BQ, UK

## Abstract

Testicular germ cell tumours (TGCTs) are the most common cancer in young men. Here we perform whole-exome sequencing (WES) of 42 TGCTs to comprehensively study the cancer's mutational profile. The mutation rate is uniformly low in all of the tumours (mean 0.5 mutations per Mb) as compared with common cancers, consistent with the embryological origin of TGCT. In addition to expected copy number gain of chromosome 12p and mutation of *KIT*, we identify recurrent mutations in the tumour suppressor gene *CDC27* (11.9%). Copy number analysis reveals recurring amplification of the spermatocyte development gene *FSIP2* (15.3%) and a 0.4 Mb region at Xq28 (15.3%). Two treatment-refractory patients are shown to harbour *XRCC2* mutations, a gene strongly implicated in defining cisplatin resistance. Our findings provide further insights into genes involved in the development and progression of TGCT.

## Abstract (web summary)

Testicular germ cell tumour (TGCT) is the most common cancer in young men. Here, the authors sequence the whole exomes of 42 TGCTs, and characterize the mutational profile of this tumour type.

## Graphical abstract

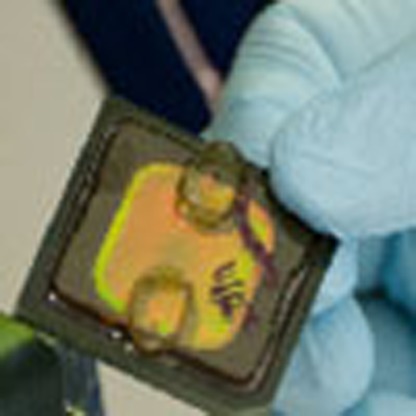

TGCTs are the most common cancer affecting young men, with a mean age at diagnosis of 36 years^1^,^2^. The main TGCT histologies are seminomas, which resemble undifferentiated primary germ cells, and non-seminomas, which show differing degrees of differentiation. Cure rates for TGCTS are generally high, due to the sensitivity of malignant testicular germ cells to platinum-based chemotherapies, however this is at the cost of an increased risk of metabolic syndrome, infertility and secondary cancer^3^,^4^,^5^. Furthermore, there are limited options for the patients who are platinum resistant, a group for whom the long-term survival rate is poor^6^.

Overall, TGCTs are markedly aneuploid with recurring gain of chromosomes 7, 8, 21, 22 and X^7^,^8^,^9^,^10^,^11^,^12^,^13^. In addition, gain of chromosomal material from 12p is noted in virtually all cases^7^,^8^,^9^, with genomic amplification and overexpression of genes in the 12p11.2-p12.1 region reported in ~10% of TGCTs^14^. *KRAS* is located in this region and has been proposed as the candidate driver^14^. Focused studies of TGCTs have identified somatic missense mutations and amplifications of the oncogene *KIT*, present in ~25% of seminomas^15^,^16^. These reported mutations are clustered in the juxta membrane and kinase encoding domains of KIT^15^,^16^. However, a study of 518 other protein kinase encoding genes failed to conclusively identify any new driver mutations^17^. Beyond these focused interrogations of specific genes, no systematic mutational analysis across all genes in a large series of TGCT samples has been reported to our knowledge.

Here we perform WES of a series of 42 TGCTs to characterize the mutational signature of these tumours and to search for additional driver mutations and pathways disrupted. Our analyses demonstrate these tumours to be relatively homogeneous in profile with a markedly low rate of non-synonymous mutations and provide some novel insights into the genomic architecture of this biologically interesting tumour type.

## Results

### Overview of TGCT mutational landscape

**Figure 1:**
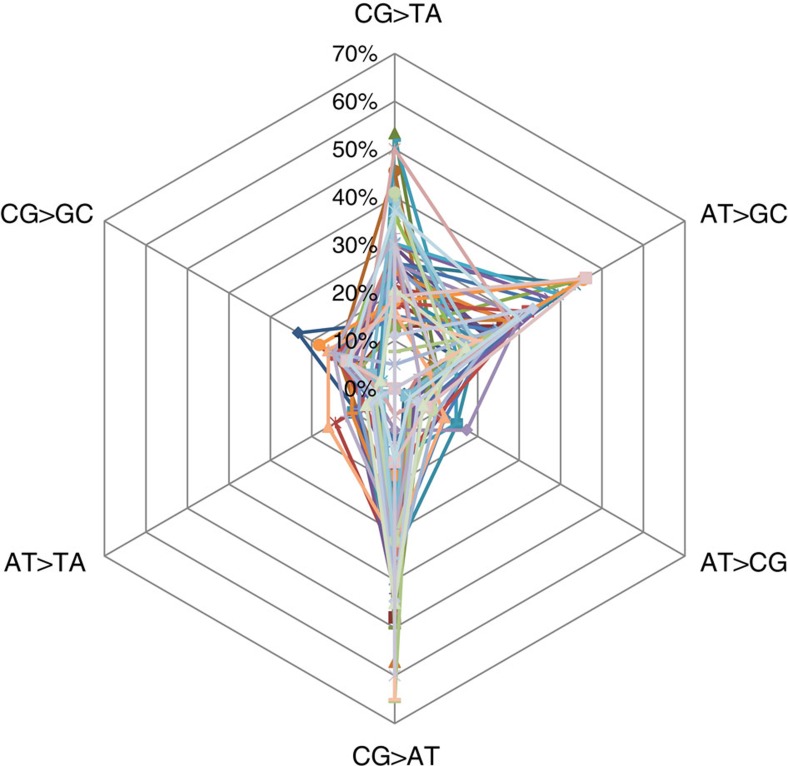
TGCT somatic SNV spectrum exome wide. Proportions are displayed for all 12 possible SNV alterations, collapsed by strand complementarity. Each line represents one of the 42 tumours.

The 42 TGCT cases comprised 16 seminomas, 18 non-seminomas, 4 mixed seminoma/non-seminoma histology and 4 tumours of indeterminant classification. Fresh frozen tumour tissue and matched germline blood samples were obtained from each patient and WES was performed on extracted DNA, achieving mean coverage of 72 × across targeted bases with 86% of targeted bases being covered at ≥20. Sequencing was conducted using Ilumina technology, with subsequent alignment, mapping and variant calling performed using Burrows–Wheeler Aligner (BWA)/Stampy/GATK/MuTect software. Across all 42 cases a total of 1,168 somatic single nucleotide variants (SNVs), and 111 small scale somatic insertion –deletions (indels) were identified, resulting in a combined total of 795 non-synonymous mutations, equating to a mean rate of 0.51 somatic mutations per Mb. By comparison, recent large-scale analysis across 27 cancer types recorded mean rates as high as 11.0 Mb^−1^ in melanoma and 8.0 Mb^−1^ in lung cancers with a mean rate across all tumour types of 4.0 Mb^−1^, some eight times higher than that seen here in TGCT (ref. 18). Indeed the mutation rate in TGCT is within the second lowest decile, only marginally greater than paediatric cancers such as Ewing sarcoma (0.3 Mb^−1^) and Rhabdoid tumour (0.15 Mb^−1^). This observation is entirely consistent with oncogenic origins of TGCT arising during embryonic development^19^. Of additional note is the high intra-patient homogeneity in mutation rate present in our data, with a s.d. of just 0.24 across the 42 tumours and the extreme lowest to extreme highest mutation rate varying by only 1 order of magnitude. This variation is low compared with the 3 orders of magnitude inter-sample variation observed for acute myeloid leukaemia, which has a comparable mutation rate^18^. Of note, there were no genes that were recurrently mutated or structural variants shared between the tumours in which the mutational rate was >2 s.d. above the mean (two tumours). The mutational spectrum of SNVs in the TGCTs was typified by an excess of CG>TA transitions (27% of SNVs), as observed in most solid tumours^18^,^20^ (Fig. 1). In addition, TA>CG transitions (23%) as well as CG>AT transversions (31%, of which the majority were C>A) were also over-represented. While C>A transversions are observed at higher proportion in lung cancers postulated to be due to exposure to tobacco carcinogens^18^, this pattern is also has also been reported in melanoma, neuroblastoma and chronic lymphocytic leukaemia^21^.

### Driver genes

**Figure 2:**
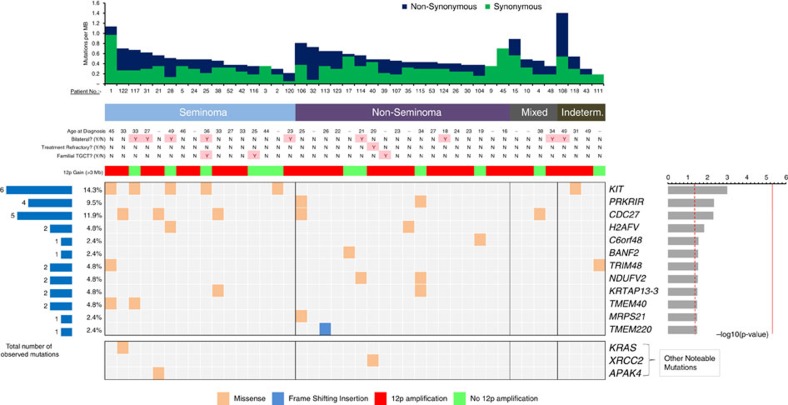
Mutated genes in testicular germ cell tumour by histological subtype. The top bars represent somatic mutation rate per sample for the 42 samples (synonymous and non-synonymous (including small-scale indels)). The genes listed on the right are mutated genes as prioritized by MutSigCV, ranked by −log_10_(*P* value) (far right), with the dotted red line denoting a significance threshold of *P*=0.05 and the solid red line a genome-wide significance threshold of 5 × 10^−6^ (see Methods). Below the top ranked genes in a separate box are other notable but non-significant mutations. Mutations by sample are depicted in the central box, with colour indicating mutation type as per the legend. The far left bars represent the absolute number of mutations observed per gene across all samples and adjacent to this is the % of samples this represents.

We used MutSigCV version 1.4 to identify genes harbouring more non-synonymous mutations than expected by chance given gene size, sequence context and gene-specific background mutation rates^18^. *KIT* was identified as the most significantly mutated gene (Fig. 2), with mutations seen in 14.3% across all TGC tumours, but predominantly found in seminomas (31.3%); a result consistent with previously reported observations^16^,^22^. All of the six *KIT* mutations we identified were in hotspot domains—five non-synonymous SNVs in exon 17 (kinase encoding domain) and one in exon 11 (juxta membrane domain). The absence of another gene ranked above *KIT* is a notable result, given our study assesses an exome-wide compliment of genes. In addition to *KIT*, a non-synonymous SNV was also observed in previously proposed TGCT driver gene *KRAS*. While *p53* mutations have been suggested to be a feature of TGCT^23^, none were observed in our data set, consistent with most recent studies^17^,^24^,^25^. We validated all *KIT/KRAS* mutations called by next generation sequencing (NGS) using Sanger sequencing of the respective exons across all samples and to ensure no additional mutations were missed. In all cases, Sanger sequencing was 100% concordant with NGS.

In addition to *KIT* and *KRAS*, there was an over-representation of mutations in cell division cycle 27 (*CDC27*) (11.9%; 5 mutations, 5 tumours) and *PRKRIR* (4 mutations, 2 tumours), neither of which have been previously reported as TGCT drivers. *CDC27* is a core component of the anaphase-promoting complex/cyclosome, a multi-subunit E3 ubiquitin ligase that governs cell cycle progression, through ubiquitination and degradation of G1/mitotic checkpoint regulators^26^. Anaphase-promoting complex/cyclosome recruits its substrates via one of the two adaptor proteins CDC20 or CDH1, overexpression of which have been linked to multiple tumours^27^,^28^,^29^. *CDC27* is downregulated in breast cancer and *CDC27* is postulated to be a tumour suppressor^30^. All of the *CDC27* mutations we identified were missense variants, characterized by a consistently low frequency of mutant allelic reads (8–14%), consistent with *CDC27* mutation being present only in a subclone of each tumour sample. Intriguingly subclonal low frequency of *CDC27* mutation has also recently been demonstrated in a colonic adenocarcinoma^31^.

### Pathway analysis

To increase our ability to identify cancer drivers and delineate associated oncogenic pathways for TGCT, we incorporated mutation data from multiple tumour types using Oncodrive-fm^32^ as implemented within the IntOGen-mutations platform^33^. The most frequently mutated pathways were those involved in metabolism (mutated in 93%), pathways in cancer (54%), endocytosis (54%) and PI3K–Akt signalling (54%). The most significantly mutated pathway was RNA degradation (14.6%), with a biased accumulation of functional mutations (fm-bias, *P*=3.8 × 10^−3^), observed across six different genes (see methods and Supplementary Table 2).

### Copy number variation

**Figure 3:**
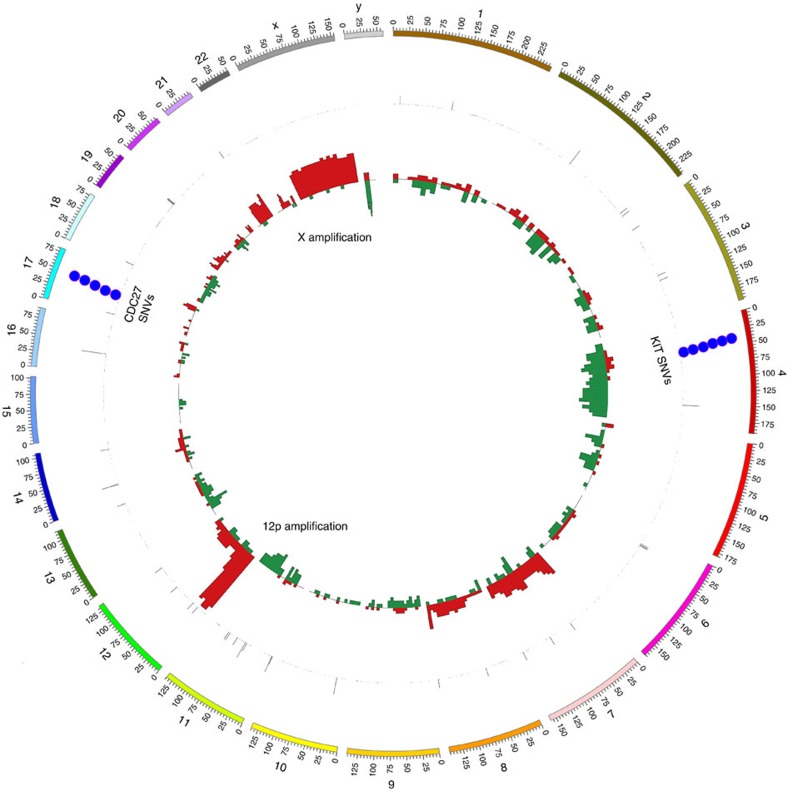
Circos Plot showing the count of SNV variants and copy number changes in the 42 tumours. Outer ring marks the count of SNV variants across all 42 samples with proposed driver SNVs as blue dots and other SNVs as black lines; inner ring marks large-scale copy number gains (red) and losses (green).

The 42 tumours were analyzed for copy number variation (CNV) using software package ExomeCNV^34^. Focal CNVs (up to 3 Mb) were identified in all tumours and large-scale CNVs (≥3 Mb) were detected in 35 (83%) tumours, (Fig. 3). Across all 42 cases the proportion of the tumour genome showing CNV ranged from 0.1 to 48.4% per genome (mean 10.8%). The most frequent large-scale chromosome abnormality was 12p copy number gain, present in 30 of the 42 tumours (71%), of which 25 were 12p isochromosomes, a result consistent with previous experimental observations^7^,^8^,^9^. The remaining 12 cases without large-scale 12p gain all showed evidence of focal copy number amplification of 12p, however, detailed analysis of these sub-regions did not reveal any recurring hotspots. Other recurring large-scale copy number changes included gain of chromosome X (16 cases, 38%) as well as gains of chromosomes 7 (*n*=15; 36%), 21 (*n*=12; 29%) and 22 (*n*=11; 26%), findings again consistent with previous studies^7^,^8^,^9^,^10^,^11^,^12^,^13^. In addition, we observed large-scale copy number deletion of chromosome Y (10 cases, 24%). We used previously generated chromosomal comparative genomic hybridization (CGH) data for 24 of the tumours^12^,^35^,^36^ to validate our large-scale CNVs for the known mutational event at 12p; concordance between NGS/CGH was 92%.

In terms of focal events three tumours (patients 115, 53 and 43) exhibited a high degree of chromosomal instability, with a 19-fold increase in focal alterations compared with the others. We assessed these cases for evidence of chromothripsis, which we defined as >20 CNVs on a chromosome single arm. While this technical definition was met for several loci, the majority of events were spread uniformly across the genome with no common hotspots across the three tumours. Excluding these three tumours we undertook an analysis of the focal alterations seen in the remaining 39 tumours to identify any recurrent patterns. Mapping the coordinates of all focal copy number events to genes, all possible gene alterations were assessed, quality filtered and ranked by frequency (Table 1 and methods). The highest ranking gene from this analysis was fibrous sheath interacting protein 2 (*FSIP2*) at 2q32.1, with seven recurring amplifications observed across six (15.3%) tumours. *FSIP2* amplifications were all 8–9 kb in length spanning a sub-region of the gene coding sequence, encompassing exons 16–17. Recent functional evidence has demonstrated that part-gene amplifications do affect gene expression levels, with an effect size comparable to that of full-gene amplification^37^. Our finding of recurrent *FSIP2* amplification is corroborated by recent high resolution SNP array data on an independent series of seminomas^38^, which documented *FSIP2* amplification in 22% of tumours. Across both studies *FSIP2* is the only gene consistently observed with focal amplification in >10% of cases. There is a strong biological basis for abnormalities of *FSIP2* being a feature of TGCTs *a priori.* The fibrous sheath is a cytoskeletal structure located in the principle piece region of the sperm flagellum. Transcription of *FSIP2* begins in late spermatocyte development with mouse model data demonstrating it to be expressed exclusively in the testis^39^. Furthermore, FSIP2 also binds to another fibrous sheath enzyme A kinase (PRKA) anchor protein 4 (AKAP4), which has been linked to male infertility^40^. Interestingly the tumour from patient 21, which harboured a *FSIP2* amplification, also carried a missense mutation in *AKAP4*.

Other focal events observed included a 0.4 Mb region at Xq28, with amplification in six cases. This region contains 18 genes, including testis expressed 28 (*TEX28*) and transketolase like gene 1 (*TKTL1*), both of which are overexpressed in the human testis^41^. *TKTL1* is hypothesized to play a role in tumour response to hypoxia with increased *TKTL1* expression correlating with poor patient outcome in many solid tumours^42^.

### Clinicopathological-molecular associations

SNV/indel somatic mutation rates between seminoma and non-seminoma cases were almost identical; 0.50 mutations per Mb and 0.49 mutations per Mb respectively. *KIT* mutations were observed predominantly in seminoma cases, as previously reported. The proportion of the genome showing CNV was elevated (+47%) in non-seminona tumours. A correlation between somatic mutational rate and patient age was seen (*r*=0.36), with the mean rate for patients aged >40 years being 0.69 compared with 0.48 for cases <40 (*P*=0.05, two-sided Student’s *t*-test). This is consistent with a model in which the majority of mutations are passenger mutations that accumulate with patient age following the early *in utero* oncogenic transformation of germ cells. Of particular clinical interest is the mutational profile of treatment-refractory TGCT, a rare subset of ~3% of patients in whom there is disease progression despite platinum-based chemotherapy. Within our cohort only one such patient, 40, had this profile of therapeutic response, so any conclusions are speculative. Accepting this caveat the mutational rate for this tumour was 0.49 Mb^−1^, a rate comparable to the overall cohort, and of the 18 SNVs identified in this patient (see Supplementary Table 1), a mutation in gene *XRCC2* (c.6T>Gp.Cys2Trp) is of particular note. *XRCC2* encodes a member of the RecA/Rad51-related protein family, which participates in homologous recombination maintaining chromosome stability and repair of DNA damage. Importantly *XRCC2* mutant animal clones show increased resistance to cisplatin through enhanced DNA repair activity^43^, and *XRCC2* germline variants have been shown to significantly associate with cytotoxic resistance in breast cancer^44^. In addition to the treatment-refractory patient in our main cohort, we also performed exome sequencing of tumour DNA from one additional platinum refractory case (germline DNA was not available, patient 109), identifying a further mutation in *XRCC2* (c.2T>Gp.Met1Arg). This additional variant had alternative allele frequency of only 4%, making it difficult to validate by Sanger. Both *XRCC2* mutations are predicted to be pathogenic on the basis of *in silico* analysis using the CONDEL algorithm (CONsensus DELeteriousness (CONDEL) score of non-synonymous SNVs, http://bg.upf.edu/fannsdb/help)^45^,^46^.

## Discussion

Our exome analysis has confirmed mutation of *KIT* and recurrent copy number gain of 12p as archetypical features of TGCT. We have also characterized the mutational signature of TGCTs, demonstrating a homogeneous profile with a markedly low SNV mutation rate, consistent with the embryonic origins of the disease. This low rate of point mutations (that is, SNVs) is contrasted, however, by frequent large-scale copy number gains, of not only 12p but also chromosomes 7, 21, 22 and X. Since our study was empowered to identify recurrent mutations having frequency of >15% (84% power), we can conclude that it is unlikely that additional high frequency driver mutations will exist.

We did, however, identify novel mutations in the probable tumour suppressor gene *CDC27*, implicating *CDC27* mutation as a potential oncogenic factor in a subset of TGCTs. Functionally *CDC27* interacts with spindle checkpoint proteins encoded by *MAD2* (ref. 47) and *TEX14* (ref. 48) genes, the latter of which resides in a linkage disequilibrium block associated through recent genome-wide association study (GWAS) with germline TGCT predisposition^49^. Interestingly three of the other TGCT GWAS risk loci contain genes also related to mitotic spindle assembly—*MAD1L1, CENPE* and *PMF1* (refs 49, 50). Collectively, such observations provide further evidence of commonality between germline and somatic TGCT pathways, a notable result given the previous precedent that *KITLG*, the ligand which binds *KIT*, is the only gene within the linkage disequilibrium block at the strongest existing TGCT GWAS risk locus (odds ratio~2.5)^51^. Aside from *CDC27*, we also observed mutations in several other genes at a frequency of <10%; at this lower frequency our study was not sufficiently powered to comprehensively evaluate the genetic mutational profile (our power to detect mutations with frequencies of 10% and 5% was only 14%).

Previous CGH studies have characterized the aneuploidy nature of TGCTs, and our findings are consistent with these analyses. We hypothesized that NGS exome data, with average probe lengths of ~200 bp, would allow identification of novel small-scale CNVs below the level detectable by CGH. We performed this analysis and identified recurring focal copy number alterations in the spermatocyte development gene *FSIP2*, a finding corroborated by previous independent orthologous study. Meta-analysis of the two experiments shows this to be significant at *P*=6.8 × 10^−9^. *FSIP2* is shown to be unique to spermatogenic cells and is hypothesized to act as a linker protein, binding *AKAP4* to the fibrous sheath^39^. Dysplasia of the fibrous sheath and mutations in *AKAP4* have both been linked to male infertility^40^,^52^, an established risk factor for TGCT^53^. The additional observation of an *AKAP4* missense mutation further implicates this pathway, although the exact mechanisms facilitating tumorigenesis remain to be elucidated. Furthermore, we observed recurrent deletion of chromosome Y, a finding that also has interesting resonance with the germline as chromosome Y ‘gr/gr’ germline deletions are linked to both TGCT predisposition and male infertility^54^,^55^. In addition, we identified a recurring focal amplification of 0.4 Mb in length at Xq28, a region encompassing 18 genes, several of which may plausibly link to TGCT. Several observations implicate chromosome X in germ cell oncogenesis, with family studies suggesting a possible X-linked model of inheritance for TGCT genetic susceptibility^56^. In addition, patients with Klinefelter syndrome (47XXY constitutional karyotype) have a 67-fold elevated risk of developing mediastinal germ cell tumours^57^.

We found no significant difference observed in the mutational rate between seminoma and non-seminoma cases. This is consistent with findings from germline genetic studies of TGCT, where no differential genotype risk has been observed between histological sub-groups^49^,^51^,^58^. This supports a hypothesis of commonality in the oncogenic pathways activated, with differentiation occurring later in the tumour formation. This hypothesis is further supported by the observation of TGCT cases with mixed pathology^59^, as well as bilateral and familial cases displaying tumours with inconsistent histological types^60^,^61^. Descriptive analysis of a single treatment-refractory patient in our cohort revealed a *XRCC2* mutation, a DNA repair gene which has been demonstrated to promote cisplatin resistance in animal studies^43^. Further analysis of one additional treatment-refractory tumour sample revealed some evidence for a second *XRCC2* mutation. Cell line studies suggest that the exceptional sensitivity of TGCTs to cisplatin is due to their inability to repair treatment-induced DNA damage, due to the low expression of DNA repair genes such as *ERCC1* (ref. 62). In addition, cisplatin-resistant embryonal carcinoma cell lines show sensitivity to poly(ADP-ribose) polymerase (PARP) inhibition, through blocking their acquired ability to repair DNA^63^. The observation of *XRCC2* mutations in our patient tumour data expands on these previous animal and cell line studies, further supporting an important role for this pathway.

To our knowledge this study represents the largest comprehensive sequencing study of TGCT conducted to date. While we have implemented strategies to accurately identify the mutational landscape of this tumour, we were only well powered to identify genes with high mutational frequency. Hence further insights into the biology of TGCT should be forthcoming through additional sequencing initiatives and meta-analyses of such data. This is likely to be especially important given the importance of probable histological subtype-specific changes, the subclonal architecture of TGCT and differences that are likely to be seen in platinum-resistant tumours.

## Methods

### Sample description

Samples were collected from TGCT patients at the Royal Marsden Hospital NHS Trust, UK. Informed consent was obtained from all participants and the study was approved by the Institute of Cancer Research/Royal Marsden Hospital Committee for Clinical Research (study number CCR2014). The samples have been previously reported in other studies^10^,^12^,^36^,^61^,^64^. Surgical specimens were snap frozen within 30 min of surgery and matched blood samples were collected at the time of surgery. Tumour samples were trimmed to remove surrounding normal tissue, and tumour cells were confirmed by histological assessment. Tumour and matched lymphocyte DNA were extracted by standard techniques^65^,^66^. Tumour samples from patients 26 and 9 were obtained post chemotherapy. Clinical characteristics of our sample cohort were representative of the broader patient population, in terms of histological sub-types, patient age, familial TGCT and response to treatment. Our series was, however, enriched for cases with bilateral disease (9/42 cases in our series compared with a frequency of ~5% in the broader patient population).

### Whole-exome sequencing

Samples were quantified using Qubit technology (Invitrogen, Carlsbad, CA, USA) and sequencing libraries constructed from 50 ng of respective normal/tumour DNA. Library preparation was performed using 37 Mb Nextera Rapid Capture Exome kits (Ilumina, San Diego, CA, USA), with enzymatic tagmentation, indexing PCR, clean-up, pooling, target enrichment and post-capture PCR amplification/quality control performed in-house, following standardized protocols as per manufacturer guidelines. Samples underwent paired-end sequencing using the Ilumina HiSeq2500 platform with a 100-bp read length. Mean coverage of 73.6 × and 69.0 × were achieved across targeted bases for tumour and normal samples, respectively. FASTQ files were generated using Illumina CASAVA software (v.1.8.1, Illumina) and aligned to the human reference genome (b37/hg19) using BWA (v. 0.5.10, http://bio-bwa.sourceforge.net/)/ Stampy (v.1.0.23) packages. PCR duplicates were removed and coverage metrics were calculated using Picard-tools (v.1.48, http://picard.sourceforge.net/). Coverage metrics demonstrated a mean of 95% of target bases achieved >10 × coverage and 86% >20 × . The Genome Analysis Toolkit (GATK, v. 3.1-1, http://www.broadinstitute.org/gatk/) was used for local indel realignment/base quality score recalibration and SNVs were called using MuTect (v. 1.1.4). Data was quality filtered using in-house FoxoG software to remove potential artefactual variants introduced through DNA oxidation^21^. FoxoG ensured variants were supported by a minimum of one alternative read in each strand direction, a mean Phred base quality score of >26, mean mapping quality ≥50 and an alignability site score of 1.0. Small-scale insertion/deletions (indels) were called using GATK.

We used MutSigCV (v.1.4) to identify genes that somatically mutated more often than would be expected by chance^18^, after first excluding common germline SNPs with minor allele frequency >25% as recorded in either dbSNP (http://www.ncbi.nlm.nih.gov/SNP/), 1000 genomes (http://www.1000genomes.org) or in our in-house data from exome sequencing of the UK 1958 birth cohort (Houlston *et al*., personal communication). In total, 33 common germline SNP variants were removed across all samples. MutSigCV was run using the standard genomic covariates of (i) global gene expression data, (ii) DNA replication time and (iii) HiC statistic of open versus closed chromatin states. We used Oncodrive-fm^32^ as implemented within the IntOGen-mutations platform^67^, using data mutation data from multiple tumour studies (http://bg.upf.edu/group/projects/oncodrive-fm.php; http://www.intogen.org/analysis/mutations/)

### Confirmation sequencing

Confirmation sequencing was performed with bidirectional Sanger sequencing of *KIT* (exons 11 and 17) and *KRAS* (exon 2) across all 84 tumour/normal samples. Primer sequences are shown in Supplementary Table 3. Mutational analysis was conducted using Mutation Surveyor (v.3.97, SoftGenetics, State College, PA, USA).

### CNV analysis

CNV analysis was conducted using the CRAN package ExomeCNV^34^, a statistical algorithm designed to detect CNV, and loss of heterozygosity (LOH) events using depth-of-coverage and B-allele frequencies (https://secure.genome.ucla.edu/index.php/ExomeCNV_User_Guide). ExomeCNV is calibrated to achieve high levels of sensitivity and specificity, with a power to detect 95% for CNVs down to 500 bp in length^34^. When recently tested using a matched tumour/normal exome data set with ~40 × coverage, ExomeCNV achieved 97% specificity and 86% sensitivity compared with results from Illumina Omni-1 SNP array^34^. To calculate CNVs, we first generated coverage files using GATK, and then used ExomeCNV to calculate log coverage ratios between matched tumour/normal samples and make CNV calls per exon. Exonic CNV calls were combined into segments using circular binary segmentation. LOH calls were made by first identifying all heterozygous germline positions per case, using Platypus (v.0.5.2) for germline variant calling. GATK was then used to create BAF files per case and ExomeCNV used to call LOH at heterozygous positions individually and at combined LOH segments.

CNV results were classified as large-scale (>3 Mb in length) or focal (<3 Mb) and filtered by coverage ratio selecting copy number gain >1.3 or loss <0.7, retaining calls with a specificity confidence score of 1.0. Focal events were analyzed by gene, mapping the coordinates of all events to gene coding start and end points to assess all possible gene alterations. Small-scale regions showing susceptibility to variable levels of coverage, that is, exact same probes frequently altered and with both copy number gain and loss, were removed to avoid false-positive associations.

### Pathway analysis

Pathway analysis was performed using Oncodrive-fm^32^ as implemented within the IntOGen-mutations platform^67^, using the 1,168 SNVs and 111 indel mutations called across the 42 tumours.

### Statistical analyses

Statistical significance of mutations were determined by testing whether the observed mutation counts in a gene significantly exceeded the expected counts based on a gene-specific background mutation rate, as implemented in MutSigCV (v.1.4). Plotted in the far section of Fig. 2 are the resulting −log10 (*P* values), with the dotted red line denoting a significance threshold of *P*=0.05 and the solid red line a genome-wide significance threshold of *P*=5 × 10^−6^. Due to the overall low frequency of mutations observed in our data set, and the way such tumour types are treated by MutSigCV, no genes were significant at the genome-wide level, not even previously known TGCT driver gene *KIT*. Power analysis was conducted using a binomial power model, based on recent methods published by the Cancer Genome Analysis group at the Broad Institute^68^, incorporating the average background somatic mutation rate specifically observed for TGCT, sample size and assuming a genome-wide significance level of *P*≤5 × 10^−6^. Significance of focal copy number events by gene was calculated under a binomial distribution. Meta-analysis was conducted using the Fisher method of combining *P* values from independent tests. Statistical analysis were carried out using R3.0.2 (http://www.r-project.org/) and Stata12 (StataCorp, Lakeway Drive College Station, TX, USA) software. Continuous variables were analyzed using Student’s *t*-tests. We considered a *P* value of 0.05 (two sided) as being statistically significant.

## Additional information

**How to cite this article:** Litchfield, K. *et al*. Whole-exome sequencing reveals the mutational spectrum of testicular germ cell tumours. *Nat. Commun.* 6:5973 doi: 10.1038/ncomms6973 (2015).

**Accession codes:** Whole-exome sequencing have been deposited in the European Genome–phenome Archive (EGA), which is hosted by the European Bioinformatics Institute (EBI), under the accession code EGAS00001001084.

## Supplementary Material

Supplementary Information Supplementary Tables 1-3

## Figures and Tables

**Table 1:** Genes with five or more recurrent copy number gains/losses.

**Gene (s)**	**Region**	**Losses**	**Gains**	**Total CNVs**
* FSIP2 *	2q32.1	2	7	9
* AK2 *	1p35.1	0	7	7
* ZNF644 *	1p22.2	0	7	7
* ENPP3 *	6q23.2	0	7	7
* MUC12 *	7q22.1	0	7	7
* AHNAK2 *	14q32.33	0	7	7
* TSPEAR *	21q22.3	0	7	7
* FLG *	1q21.3	1	6	7
*AK056431*	1q21.3	1	6	7
*HCFC1, TMEM187, MIR3202-1, IRAK1, MIR718, MECP2, OPN1LW, TEX28, OPN1MW, TKTL1, FLNA, EMD, AK307233, RPL10, SNORA70, DQ570720, DNASE1L1, TAZ*	Xq28	0	6	6
* CHRND *	2q37.1	0	6	6
* CTAGE9 *	6q23.2	0	6	6
* MUC5B *	11p15.5	0	6	6

CNV, copy number variation.

Focal CNVs included are defined as <3 Mb in length. See methods for further details on quality filters applied.
