# Supplementary material for: Whole-exome sequencing reveals the mutational spectrum of testicular germ cell tumours
Source: Nat Commun. 2015 Jan 22;6:5973. doi: 10.1038/ncomms6973 (PMC4338546; doi:10.1038/ncomms6973)
Supplement: Supplementary Information — Supplementary Tables 1-3 [file ncomms6973-s1.pdf]

Supplementary Information

Supplementary Table 1: Mutations observed in treatment refractory patient:

CHROM	POS	REF	ALT	Gene	exon	hgvs	class
1	70555500	A	G	LRRC7	Ex23of25	c.4429A>Gp.Lys1477Glu	NSC
2	44051563	T	C	ABCG5	Ex8of13	c.913A>Gp.Thr305Ala	NSC
2	111399042	C	A	BUB1	Int21/22	c.2626-1G>T	ESS
3	175165091	A	G	NAALADL2	Ex6of14	c.1165A>Gp.Lys389Glu	NSC
5	66461444	C	A	MAST4	Ex29of29	c.6437C>Ap.Pro2146Gln	NSC
7	100683053	A	G	MUC17	Ex3of13	c.8356A>Gp.Thr2786Ala	NSC
7	100683036	T	C	MUC17	Ex3of13	c.8339T>Cp.Ile2780Thr	NSC
7	152373159	A	C	XRCC2	Ex1of3	c.6T>Gp.Cys2Trp	NSC
7	100349919	T	C	ZAN	Ex13of46	c.2191T>Cp.Ser731Pro	NSC
11	71946892	C	A	INPPL1	Ex25of28	c.2741C>Ap.Ser914X	SG
12	11461675	C	G	PRB4	Ex3of4	c.242G>Cp.Arg81Pro	NSC
15	41192690	C	A	VPS18	Ex4of5	c.1674C>Ap.Tyr558X	SG
18	23731828	C	A	PSMA8	Ex3of7	c.254C>Ap.Thr85Asn	NSC
19	17392967	G	T	ANKLE1	Ex2of9	c.164G>Tp.Gly55Val	NSC
19	4429600	C	A	CHAF1A	Ex9of15	c.1770C>Ap.Asp590Glu	NSC
19	18547504	C	G	ISYNA1	Ex5of11	c.599G>Cp.Arg200Pro	NSC
19	44352666	G	A	ZNF283	Ex7of7	c.1913G>Ap.Cys638Tyr	NSC
19	56663216	A	G	ZNF444:AC024580.1	Int3/4:Ex1of2	c.297+4639A>G:c.35T>Cp.Val12Ala	INTR:NSC

Supplementary Table 2 – Mutated pathways

2A) Mutated pathways ordered by frequency of observed mutations. Fields as calculated by IntoGen, see URL http://www.intogen.org/ for documentation

pathway	fm-bias	found/studied
Metabolic pathways	0.027	38 / 42
Pathways in cancer	0.334	22 / 42
Endocytosis	0.894	22 / 42
PI3K-Akt signaling pathway	0.013	22 / 42

2B) Mutated pathways ordered by significance (fm-bias). Fields as calculated by IntoGen, see URL http://www.intogen.org/ for documentation

pathway	fm-bias	found/studied
RNA degradation	3.815E-3	6 / 42
Acute myeloid leukemia	0.013	9 / 42
PI3K-Akt signaling pathway	0.013	22 / 42

Supplementary Table 3 – Primer sequences used for validation of known driver mutations in *KIT* and *KRAS*

Gene_Exon_Direction	Primer sequence (5' to 3')
KIT_Ex10-11_F	ATGGGCTGTGAGTTGGGAG
KIT_Ex10-11_R	TCAGGTGGAACAAAACAAAGG
KIT_Ex17_F	TGTGAACATCATTCAAGGCG
KIT_Ex17_R	AAATGTGTGATATCCCTAGACAGG
KRAS_Ex2_F	TTTGTATTAAAAGGTACTGGTGGAG
KRAS_Ex2_R	TGAAACCCAAGGTACATTTCAG
